# Generating Multi-Functional Pulse Ingredients for Processed Meat Products—Scientific Evaluation of Infrared-Treated Lentils

**DOI:** 10.3390/foods12081722

**Published:** 2023-04-20

**Authors:** Darshika Pathiraje, Janelle Carlin, Tanya Der, Janitha P. D. Wanasundara, Phyllis J. Shand

**Affiliations:** 1 Department of Food Science and Technology, Wayamba University of Sri Lanka, Makandura 60000, Gonawila, Sri Lanka; dpathiraje@wyb.ac.lk; 2 Department of Food and Bioproduct Sciences, University of Saskatchewan, Saskatoon, SK S7N 5A8, Canada; janitha.wanasundara@agr.gc.ca; 3 Pulse Canada, Winnipeg, MB R3C 0A5, Canada; jcarlin@pulsecanada.com (J.C.); tder@pulsecanada.com (T.D.); 4 Agriculture and Agri-Food Canada, Saskatoon Research and Development Centre, Saskatoon, SK S7N 0X9, Canada

**Keywords:** meat products, pulses, lentil, infrared treatment, fresh meat color, lipid oxidation

## Abstract

In the last decade, various foods have been reformulated with plant protein ingredients to enhance plant-based food intake in our diet. Pulses are in the forefront as protein-rich sources to aid in providing sufficient daily protein intake and may be used as binders to reduce meat protein in product formulations. Pulses are seen as clean-label ingredients that bring benefits to meat products beyond protein content. Pulse flours may need pre-treatments because their endogenous bioactive components may not always be beneficial to meat products. Infrared (IR) treatment is a highly energy-efficient and environmentally friendly method of heating foods, creating diversity in plant-based ingredient functionality. This review discusses using IR-heating technology to modify the properties of pulses and their usefulness in comminuted meat products, with a major emphasis on lentils. IR heating enhances liquid-binding and emulsifying properties, inactivates oxidative enzymes, reduces antinutritional factors, and protects antioxidative properties of pulses. Meat products benefit from IR-treated pulse ingredients, showing improvements in product yields, oxidative stability, and nutrient availability while maintaining desired texture. IR-treated lentil-based ingredients, in particular, also enhance the raw color stability of beef burgers. Therefore, developing pulse-enriched meat products will be a viable approach toward the sustainable production of meat products.

## 1. Introduction

It is necessary for people to have access to sufficient food that is safe, nutritious, and affordable and that is produced using environmentally responsible and sustainable production systems. Several dietary surveys have also shown the widespread prevalence of the suboptimal intake of protein and micronutrients for certain segments of the world population [1,2,3,4]. The EAT-Lancet Commission [5] reports that “nearly one billion people in the world lack sufficient food and many more consume an unhealthy diet that contributes to premature death and morbidity” and that major dietary changes will be needed to achieve healthy diets by 2050, including a “greater than 100% increase in the consumption of healthy foods such as legumes, vegetables, fruits, and nuts”. Among the animal protein sources in the diet, meat is a whole food and can be converted into several meat-based products. Meat is a major source of both energy and high-quality protein and micronutrients, including B12 and D vitamins, iron, zinc, and selenium.

Proteins in the diet and their sources have evolved over time. In the beginning, slaughtered animals were used as entire carcasses or whole meat cuts, and this eventually led to their use in processed meat products to achieve full utilization of the meat and byproducts such as organs and other tissues. Prolongation of the shelf life as well as minimizing wastage through the ability to use various animal carcass parts in processed meat provides a significant advantage in the food basket of many civilizations and cultures. Such products are still popular because of their perfect merge with safe, tasteful, and convenient ready-to-eat protein-rich food in today’s fast-paced lifestyle. Owing to the dominance of existing fast-food practices, the global market demand for processed meats grew at a compound annual growth rate (CAGR) of 6.5% (2017–2021) and is expected to rise 6.3% (2022–2032), reaching a total of USD 605.14 billion in 2032 [6].

As the world’s population grows, so does the demand for animal protein in which a considerable portion is satisfied with processed meat products. Some evidence suggests that the intake of red meat and extensively processed meat is associated with an increased risk of chronic illnesses such as cardiovascular diseases, type 2 diabetes, and some forms of cancer [7,8]. Therefore, in the context of minimizing the negative impacts on the environment, public health, and animal welfare, the meat processing industry is focusing on blending plant-based ingredients into conventional meat products. According to the EAT-Lancet report, eating more plant-based meals and fewer foods derived from animals is beneficial for both human health and the sustainability of the planet and our environment.

The reformulation of conventional meat products incorporating plant-based ingredients offers the meat industry several opportunities that benefit both the processor and the consumer. These include developing healthier products with more natural, functional ingredients; reducing waste generation while maximizing the total carcass utilization; diversification of product categories with new and improved hedonics; and lowering production costs to increase consumer affordability. The hundreds of different types of comminuted meat products available worldwide vary based on the method of manufacturing and composition of ingredients used in them. In an 1899 book on sausage making, Duff [9] listed various fillers and binders, including bread or crackers, corn starch, potato and other flours, and boiled rice, that were added to sausage for palatability and economic reasons. Processors today expect more from the plant-based ingredients that are chosen for particular functions in meat products and have made available ingredients through a wide array of processes to modify/optimize their functionalities. Among the protein-rich plant ingredients, soy-based ingredients have been around for over 60 years [10] and are widely used by the meat processing industry. In more recent times, several studies examined the use of pulses and pulse flours in various forms in meat products and discovered their effectiveness in improving the binding capacity of fat and water, fat-emulsifying ability, viscosity, and adhesiveness of meat batters [11,12,13,14]. From a nutritional standpoint, among plant-based ingredients, pulses provide high quantities of proteins and micronutrients and are a suitable candidate as meat substitutes.

According to Vatanparast et al. [15], Canadians’ overall diet quality improved when they consumed 100% more plant-based meat substitutes and 50% less red and processed meat, but there was a significant drop in their intake of protein, zinc, and vitamin B12. Thus, excluding meat and meat products from the diet may result in essential nutrient deficits. There are no unique food alternatives to meat or meat products that have the same nutritional profiles; therefore, it is desirable to focus on strategies that minimize the environmental impact of meat production and ways to reduce excessive meat consumption, without compromising nutritional quality of the diet.

Utilizing pulses and pulse flours in meat product formulations presents some challenges that need to be addressed at the ingredient and/or product manufacturing stage. Pulses can bring in minor compounds that are nutritionally undesirable, incompatible techno-functional properties, off-flavors, and prooxidants that hinder their application in meat products. Among the various thermal treatments of pulses to improve their usability as ingredients for various food products, infrared (IR) heating has been under scientific investigation over the last decade to overcome these limitations, and the findings appear promising [16,17,18]. IR treatment is a short-time heating process that employs electromagnetic radiation in the IR region [19].

The aim of the present review is to bring together the literature on the addition of pulse-based ingredients to comminuted meat products and, in particular, those of IR-treated pulse ingredients in meat processing. The studies described in this review were from author collections and also found through database searches of Web of Science using search terms such as “pulse, lentil, pea, bean” and “meat, beef, chicken, pork, sausage, burger, patty, meatball” and “infrared, micronization, heat-treated” over the last 20 years. Taking into consideration all the pertinent research conducted on the application of IR-treated pulses in meat products, more than 80% has focused on lentil-derived products. Therefore, this review emphasizes the contribution of IR-treated lentils on the techno-functional and sensory aspects of comminuted meat products. 

## 2. Comminuted Processed Meat Products

A common definition of processed meat is “meat that has been transformed through salting, curing, fermentation, smoking, or other processes to enhance flavor or improve preservation” [7]. Processed meat products are expanding in prominence in the food industry around the world, bringing growth opportunities to the industry and a diverse range of convenient and safe meat products to the consumer. There are hundreds of processed meats, each with its own standards of identity. Nonetheless, many of these products have great similarities due to the processing technologies employed. Among these, two broad categories can be identified based on the structural integrity of the meat utilized in their production: whole muscle and comminuted (i.e., that with reduced meat particle size).

Whole muscle products are made from large pieces or whole intact cuts of meat that are often molded and primarily seasoned, heat-processed, and smoked, while comminuted meat products are made from small pieces of meat that have been ground, minced, or chopped and often include a combination of meat and non-meat components [20]. The earliest recognizable reference of this type of processed meat product is found in a Greek play titled “The Orya,” or “The Sausage,” written about 500 B.C. [21], and historical evidence suggests that sausage has been an important item in man’s diet since about 3500 years ago, most likely a way of preserving meat. The introduction of ingredients such as salt and various flavorings to these products is lost in antiquity, but choices of non-meat ingredients continue to evolve as will be described later.

One of the benefits of manufacturing comminuted meat products is that it allows for the utilization of lower-value meat, which can then be turned into even more valuable products. Meat product manufacturers use meat trimmings from inexpensive parts of the carcass, such as the head and cheeks, as well as mechanically separated meat (MSM), in the formulation of comminuted meat products. MSM is a cost-effective method of utilizing the last traces of meat from the carcass. Therefore, the manufacturing of comminuted meat products enables optimal usage of the animal carcass, minimizing waste generation in the meat processing industry. 

Comminuted meat products can be further subdivided into formed ground or emulsion-based meat products based on the degree of comminution that the meat has gone through [22]. Comminuted meat (with varying sizes of particles) that has been molded into a specific shape or size is often considered a formed product. Formed meat products such as patties, meatballs, and nuggets account for a considerable portion of total meat consumption and are especially important in the food service industry. For example, Technomic reports [23] that 64% of beef purchased by food service operators in the United States is in ground form, such as burgers.

Emulsion-type meat products, such as frankfurters, wieners, and bologna sausage, are made with a viscous mass of finely chopped ground meat that is composed of muscle and fatty tissues. These meat mixtures (batters) are relatively complex systems and have many properties of an emulsion [24]. The dispersed phase of the meat emulsion is a multi-phase medium that consists of solid fat particles, liquid fat droplets, and air bubbles. The continuous phase comprises water and a wide variety of other components, including water-soluble and water-insoluble proteins, connective tissues, salt, and carbohydrates [25]. This multi-phasic system is stabilized by the strong protein gel resulting from denatured proteins of the continuous phase during heat treatment in the presence of salt and added water. Heat denaturation exposes hydrophobic regions of these proteins, thus leading to the formation of hydrophobic interactions, resulting in the aggregation of proteins in which fat globules are immobilized and physically entrapped [26]. As heating progresses, water may be expelled from the system, and if the protein matrix is insufficient, there is coalescence of fat droplets, indicating partial breakage of the emulsion. This is but one of many indicators of product quality that can be affected by the ingredients added.

The overall quality of meat products can be described as a set of properties that together identify what consumers appreciate about a product at purchase and/or consumption. Therefore, the quality traits of meat products are generally those associated with human sensory perception: appearance, including color, shape, and size; taste/flavor; texture; juiciness; and odor. Other than those traits, the quality of meat products is also expressed as freshness or wholesomeness, which relates to the perception that they are safe to eat in terms of being free of pathogens, parasites, toxins, or allergens. The selection of suitable non-meat ingredients for a particular meat application necessitates an understanding of the required functionalities. For example, the quality of emulsion-based meat products is determined by the stability of the emulsion, nutritional composition, and palatability aspects as perceived by the consumers. For burgers or meatballs, the meat particle size is often coarser with less protein extraction desired during mixing, but the need for adequate water and fat-holding is still important to maintain palatability. Thus, high-quality meat products require the application of suitable processing technologies along with meat and non-meat ingredients that offer the required functionalities.

The application of non-meat ingredients provides opportunities to enhance the overall quality of processed meat products because of the desirable functionalities they provide to the final product. Generally, food standards of the country regulate the levels of non-meat ingredients in the meat products that are produced or marketed in their jurisdictions. Table 1 shows the compositional requirements for some meat products in Canada, which relies heavily on protein content.

## 3. Pulses as Ingredients for Processed Meats

### 3.1. Composition and Nutritional Value

Pulses are dry edible legume seeds consumed in many regions of the world as part of a staple diet and have a long history dating back approximately 11,000 years. In general, pulses are rich in protein, slowly digestible carbohydrates, dietary fiber, and a variety of micronutrients such as selenium, iron, vitamins E and A, folate, niacin, and thiamine [28]. Table 2 elaborates on the chemical composition of three commonly grown pulses: dry peas, lentils and chickpeas. The mature pulse seed consists of the seed coat, the cotyledons, and the embryo. The seed coat, also known as the hull, comprises between 7 and 15% of the total seed mass. About 85% of the mass of a seed is made up of the cotyledon, while the embryo is 1 to 4%. The seed coat of pulses consists mostly of (60–90%) non-starch polysaccharides [29]. The seed cotyledons, where the majority of the nutrients are stored, contain carbohydrates (starch and non-starch polysaccharides) and proteins together with cell wall polysaccharides and micronutrients [30,31].

From a nutritional standpoint, pulses are rich in macronutrients and micronutrients. Thus, pulses in various forms have been utilized in the reformulation of meat products as binders or to enhance their nutritional and healthy features or sensory qualities. The protein, starch, and fiber contents of pulses make them great binders as these biopolymers form complex gel networks with meat proteins [33]. Among the micronutrients of pulses, a number of naturally occurring bioactive chemicals such as enzyme inhibitors, lectins, oligosaccharides, oxalates, phytic acid, and phenolic compounds have been reported. On one hand, scientific studies show that these compounds are linked to a lower risk of several degenerative diseases due to their hypocholesterolemic, anti-cancer, anti-atherosclerotic, and anti-oxidative properties. Some of these compounds may act as prooxidants and impart unpleasant flavors by limiting their applications in food products [34]. A number of organizations, including the World Cancer Research Fund International (WCRFI), the American Institute of Cancer Research (AIDR), and Health Canada (HC), recommend the inclusion of pulses in the diet to lower cancer risk [35,36]. When considering the blending of pulses into meat products, the abovementioned characteristics become great strengths as well as challenges. Application of some processing technologies and pre-treatments is helpful to overcome or minimize the impact of the negative aspects of pulses.

### 3.2. Pulse Processing

**Figure 1:**
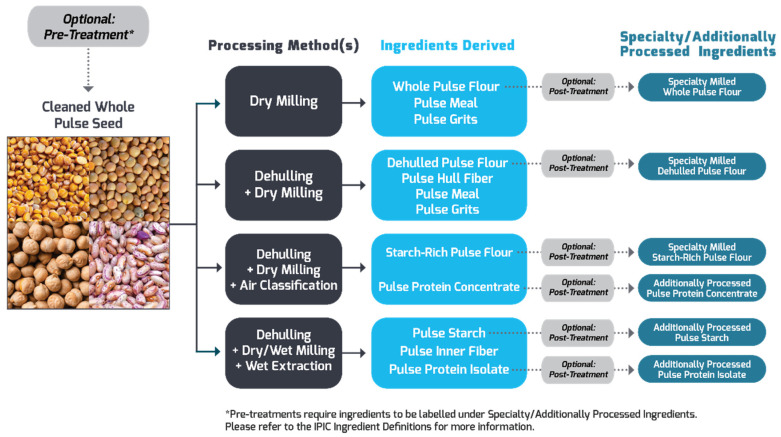
Outline of processing steps of pulses involved in the production of different pulse-derived ingredients (as defined by the International Pulse Ingredient Consortium [39]).

Pulses undergo several processes before they are used in food formulations. A summary of pulse processing into various products is shown in Figure 1. A detailed understanding of the processing of a specific pulse ingredient would be beneficial since every stage may impact the chemical composition, nutritive value, and functionality of the derived pulse product. Tannins, for example, are mostly located in the seed coat of pulses; therefore, the physical removal of the seed coat by dehulling lowers about 68 and 99% of tannin content in a seed [37]. In addition, depending on the method used for protein isolation, the types of protein and their amounts in the recovered fractions change; therefore, the functional properties differ as demonstrated for green peas and chickpeas by Chang et al. [38]. The findings of their study showed that the purity of the legumin and vicilin proteins of these fractions was greater than 80% and 90%, respectively. Vicilin proteins have stronger solubility and emulsification characteristics but lower denaturation temperature than legumin proteins owing to their smaller molecular weight, less rigid conformational structure, and lower disulfide bond content than legumin. Accordingly, it is evident that the processing can yield ingredients with different chemical compositions and techno-functional properties.

### 3.3. Pulses and Pulse-Derived Ingredients for Use in Processed Meat Products

The most explicit use of pulses in meat products is as an extender or binder/filler. Extenders and binders serve many purposes in meat products, mostly through their effects on formulation costs, improving nutritional value and benefitting processing parameters such as the viscosity and adhesiveness of meat batters and enhancing product quality by retaining more liquid (oil and water), texture, flavor, mouthfeel, and appearance [11,12,13,14]. Pulse ingredients are distributed into the meat matrix by mixing them directly into the meat during chopping and emulsification. Some recent research shows that they may also be made into pre-emulsions that utilize a high-protein pulse ingredient to stabilize a lipid emulsion before incorporation into a meat product [40]. Surface application in marinades followed by massaging may also be practiced [41]. However, the application of pulse ingredients in meat products might have both benefits and limitations depending on their constituent compounds and processing conditions under which they are used. Table 3 summarizes the potential benefits and limitations of incorporating pulses in meat products.

#### 3.3.1. Pulse Flours

The water and oil retention properties of the starch, protein, and fiber constituents of pulse flours lend to their use as a binder in food systems. In products such as patties and burgers, pulse flours trap water, fat, and other substances and form complex gel networks with meat proteins when heated (42). It has been shown that pulse flours enable high liquid retention in meat systems, thereby lowering cooking losses, improving the texture, and increasing the product yield. One particular advantage that pulses have as an ingredient in meat products is their native starches will gelatinize at normal meat thermal processing temperatures (generally ranging between 65 °C and 100 °C [40,41,42]), thus contributing greatly to the water-holding capacity. Argel et al. [43] reported that the addition of 0.8 to 1.5% chickpea, lentil, green pea, and bean flour (and 10 to 30% water) to pork patties increased the cooking yield to 87 to 91% (control value of 76%) where the type of pulse presented as a considerable factor for the cooking yield. The cooking yield of the reformulated patties improved with increasing the substitution levels, and at all substitution levels, these patties produced higher cooking yields than the commercial ones. Pork patties made with bean, lentil, and green pea flour exhibited greater cooking yield than those made with chickpea flour at the lowest level of replacement (0.8%). It was explained that the differences observed among different pulses might be due to the compositional differences of the pulse flours. Among these pulses, chickpea flour had the lowest contents of protein and fiber but the highest fat content, which could contribute to a lesser water-binding ability than other flours.

#### 3.3.2. Pulse Proteins

Lean (skeletal muscle) and fat components are often used in meat product formulations, where protein and fat from skeletal muscles make up the majority of the composition next to water. The lean meat component of the formulation is responsible for fat emulsification, product structure, water binding, and end product color [33]. Plant proteins, such as those from pulses, can substitute for the lean meat component in these products due to their functional relevance. They provide fat emulsification, gelation, structure formation, and textural integrity of the meat products [33]. Legumin and vicilin are globulin proteins of pulses that are responsible for heat-induced gelation and lead to the formation of a three-dimensional structure [44]. The heat-induced gel formation ability is maximized when the ratio of vicilin to legumin is increased [45], but it may change with the type of pulse. Proteins found in pulses also have the capacity to interact, bind, and retain water and oil [46,47] in emulsions, attributed to protein–water (ion–dipole, dipole–dipole, and hydrostatic bindings) and protein–lipid (hydrophobic) interactions. As the protein purity increases, the number of these interactions increases, resulting in more fat and water retention in emulsions [48].

#### 3.3.3. Pulse Fibers

Studies have shown that adding pea fiber to meat products increases the cook yield and water retention and reduces shrinkage [49,50]. These effects are the result of an improved capacity for the myofibrillar gel matrix to entrap water due to fiber’s absorptive ability [50,51]. Heat-induced myofibrillar gel formation provides a continuous three-dimensional gel matrix that physically entraps the added fiber [52]. Xu [53] evaluated the impact of fiber fractions from yellow peas and red lentils on low-fat pork bologna and found that they may also be used to increase the total dietary fiber content without compromising their consumer acceptability. However, some studies found that fiber addition resulted in products becoming softer and less juicy on consumption [49].

### 3.4. Bioactive Compounds of Interest in Pulses and Pulse-Derived Ingredients

Bioactive chemicals are often non-nutritive components of food that are present in smaller quantities than macronutrients. Pulses too contain an abundance of bioactive compounds that are not considered nutrients. Phenolic compounds, enzymes, oligosaccharides, and resistant starch are among the bioactive components commonly found in pulses, which makes them suitable for application in a wide range of food products.

#### 3.4.1. Non-Enzymatic Bioactives

In a recent comprehensive review, Matallana-González et al. [54] pointed out that pulses, as a good source of many different types of antioxidant compounds, may have important health benefits such as preventing cardiovascular diseases and cancer and having neuroprotective capabilities. Dietary antioxidants are a complex mixture of hydrophilic and lipophilic compounds that are abundant in foods of plant origin. Pulses contain both water-soluble (organic acids and phenolic compounds) and lipid-soluble antioxidants (tocopherols and carotenoids), as well as antioxidative minerals such as zinc and selenium.

Among the antioxidant compounds found in pulses, phenolic compounds have a significant role in delaying the oxidative degradation of meat products. Due to their redox characteristics, phenolic compounds are capable of functioning as hydrogen donors, reducing agents, and singlet oxygen quenchers [55], thereby controlling lipid, color, and flavor oxidation. Flavonoids, tannins, and phenolic acids make up the majority of the polyphenolic compounds found in pulses. Both the cotyledon and the seed coat have phenolic compounds which are found in higher concentrations in the latter [18]. The color of the seed coat is linked to the types of phenolic compounds of pulse seeds, whereby dark-colored seed coats have higher phenolic contents than light-colored pulses [56,57]. With respect to lentils, the green seed coats have higher levels of water-soluble phenolics compared with grey and brown [58].

Although bioactive compounds act in beneficial roles, these compounds in raw pulses can also have negative nutritive properties at certain concentration levels [59,60,61]. They have very different chemical properties and biological effects, and their concentrations can differ significantly from pulse to pulse and variety to variety, further influenced by the processing method applied [62,63]. However, it follows that the use of processing methods such as soaking and germination [64,65,66] or thermal processing [64,67,68] can reduce the level of antinutrients in pulses. Furthermore, since pulse ingredient addition levels in meat products are relatively low, the negative impact of these bioactive compounds on human nutrition from the consumption of pulse-added meat products may be minor.

#### 3.4.2. Endogenous Enzymes

Pulses, as with all seeds, contain enzymes such as superoxide dismutase and glutathione reductase, which provide protection against the oxidation of meat [18]. These enzymes are relatively heat-stable [18], making them useful in heat-treated food applications. On the other hand, lipoxygenase and peroxidase are oxidative enzymes present in pulses. Lipoxygenase catalyzes peroxidation of polyunsaturated fatty acids that contain one molecular oxygen with cis, cis-1,4-pentadiene structure such as in arachidonic (C20:4 n-6), linoleic (C18:2 n-6), and linolenic (C18:3 n-3) acids [69,70]. Even though the precise role of oxidative enzymes in pulses is not known, it may include fatty acid peroxidation in membranes or storage lipids, synthesis of growth regulators in response to pathogens, and nitrogen storage [71]. The activity of oxidative enzymes is necessary for the plant’s defense against pathogens, but the same activity may be negative in the food environment. Lipoxygenase mediates the conversion of polyunsaturated fatty acids to aldehydes and alcohols, which are the primary contributors to off-flavor in pulse-based products [72,73].

### 3.5. Sensory Properties of Pulses to Consider When Formulating Meat Products

Consumers often choose foods based on their sensory appeal including appearance, taste, flavor, and texture. Several studies have shown that pulses are less acceptable because of their natural sensorial properties, especially to those consumers who are not familiar with pulses. In a recent review, Chigwedere and colleagues [74] identified that beany and bitter were the most common olfactory and basic sensations, while astringent and spicy were the most common trigeminal sensations reported for pulses and pulse-derived ingredients. The unacceptable flavor profile of pulses is associated with volatile off-flavor compounds related to the presence of aldehydes, alcohols, ketones, acids, pyrazines, and sulfur compounds [75]. These compounds are either inherent to pulses or produced during harvesting, processing, and storage [76,77]. The off taste in pulses has also been associated with the presence of saponins, phenolic compounds, and alkaloids. However, very limited investigations have been conducted into the identification of off-flavor compounds in relation to their impact to the overall perception of pulses and their products. Flavor profiles of meat products depend on the inherent meat flavor in combination with that of salt, curing agents, spices, spice extractives, and other added ingredients. It will be easier to formulate with pulses or pulse-derived ingredients that have a more neutral flavor, but of course, any negative effects on palatability will depend on addition level, amount of added water, lipid levels and meat species, etc. 

Color is another attribute that may be affected when incorporating pulse flours and other pulse-derived ingredients into meat products. The use of some pulse flours in meat products has had a considerable impact on their lightness, redness, and yellowness scores. Dzudie et al. [78] observed that adding chickpea flour (7.5–10%) to beef sausages increased yellowness. According to Pietrasik and Janz [79], the color of ultra-low-fat pork bologna was unaffected by wheat or barley flours; however, lightness (a* value) was higher when produced with either 4% pea starch or wheat flour [80]. These findings show that binders have varying effects on the color of meat products, the degree of which depends on the binder and the amount used, as well as the meat product formulation. For example, some specialized textured pulse protein products have colorants added to better mimic the color of meat. Likely, other ingredients or changes in formulations may minimize any negative impacts on the color of resulting products. Consumer evaluation (n =180) of the color acceptability of sausages with 4 to 8% added whole lentil flour showed that the color of sausages did not impact the overall acceptability [81].

### 3.6. Thermal Treatments to Improve Pulse Flour Characteristics

As shown in Table 3, native characteristics and properties of pulses are not always beneficial when using them as ingredients in various food formulations. Untreated pulse ingredients contain minor amounts of several compounds and enzymes, some with undesirable nutritional effects, anti- or pro-oxidant activities, positive or negative aesthetic, taste, and techno-functional properties. In order to maximize the benefits of pulses in meat products, the negative effects of these components must be lowered, mostly by pre-treatment of the seed source or the ingredient. Various types of treatments have been employed to transform pulses into better ingredients. A few examples are boiling, roasting, germination, and fermentation, which are common across pulse-heavy food cultures and culinary traditions. At industrial-level food processing, heat treatments studied for pulses can be categorized as dry (microwave heating, extrusion cooking, etc.) or wet (boiling, canning, IR heating, etc.) depending on whether water or steam is present. One of the benefits of heat-treated pulses is the inactivation of heat-labile compounds such as trypsin inhibitors and hemagglutinins that pose undesirable nutritive effects, thereby increasing the nutritional value and quality [82]. Furthermore, heat treatment of pulses is well recognized to minimize off-flavors and hence improve the sensory quality of pulse-based products [76]. Therefore, products formulated with heat-treated pulses may have higher consumer acceptance than untreated pulses. On the other hand, heat treatment, particularly wet heating, can also cause losses of vitamins, minerals, and other water-soluble nutrients [83]. Some phenolic compounds can also be affected by heat treatment and dry heat at high temperatures generating new compounds such as Maillard browning products [84].

In pulse–meat combined product systems, the thermo-incompatibility between pulse and meat proteins is a technical challenge when incorporating pulse proteins with meat [85]. Even though processed meats are usually cooked to a final temperature of >65 °C to develop taste and texture and to make them microbiologically safe, these temperatures are not high enough to denature most plant proteins. This makes it more difficult for animal and plant proteins to interact, which is necessary to create a viscoelastic composite material framework [85]. However, prior heat treatments may facilitate the dissociation of protein subunits and partial structural unfolding [85]. As a consequence, preheat-treated pulse proteins will have improved functional capabilities such as emulsifying and water- and fat-binding in meat products. In general, heat treatment can be advantageous, particularly in deactivating oxidative enzymes, and may serve to improve the functional characteristics of pulse flours or proteins in formulations.

Over the past decade, research has focused on infrared (IR) heating as one of the thermal treatments for pulses to increase their suitability as ingredients for different foods. IR treatment is a rapid heating technique that employs IR electromagnetic radiation. IR heating is considered an advanced thermal process with reported benefits such as environmental friendliness and homogeneity of heating with low energy consumption. Thus, IR heating could serve as a viable treatment that can be used in pulse processing as a pre-treatment to enhance their functional properties.

## 4. Infrared (IR) Heat Treatment as a Pre-Treatment for Pulse Ingredients

IR heat treatment has been referred to as IR micronizing or micronization, which is a dry thermal process patented by the Micronizing Company UK. The name of the company comes from the unit of measurement of infrared wavelengths [86]. IR is electromagnetic radiation that lies between the visible and microwave regions of the electromagnetic spectrum. IR radiation is particularly efficient in generating heat inside absorbent materials by causing the component molecules to vibrate at a frequency of 60,000–150,000 MHz [87]. Intermolecular friction caused by this vibration causes rapid internal heating and a rise in water vapor pressure. Therefore, IR heating is identified as a “short-time, high-temperature” cooking process. For example, a surface temperature of 100 °C is obtained in 35 s in cereal grains [86]. IR technology can be successfully applied by industry due to its high energy efficiency, less water consumption, environmental friendliness, and homogeneity of heating compared with conventional heating [88]. Aboud et al. [89] provide a review of the mechanism and uses of IR heating in the food industry. 

### 4.1. Equipment for IR Processing

**Figure 2:**
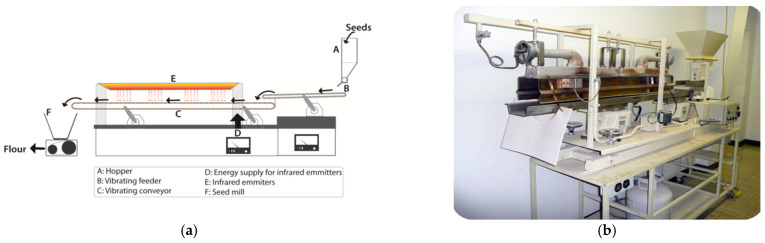
A schematic diagram (**a**) and an image (**b**) of a laboratory scale infrared heat treatment system. (Adapted with permission from Pathiratne [17] and Der personal collection, respectively).

IR ovens have a modular design that allows them to be easily fitted into most production lines, occupy less floor space than convection ovens, and require less maintenance. Figure 2 shows a laboratory-scale IR-heating system developed for the processing of grains, consisting of a vibratory conveyor and a vibratory feeder to turn the seeds frequently as they pass beneath the IR emitters [90]. IR ovens are equipped with either electric or gas-fired IR emitters, producing IR radiation in the range of 1100–2200 °C and 343–1100 °C, respectively [89]. The typical infrared wavelengths for industrial applications are between 1.17 and 5.4 µm, which correspond to temperatures between 260 and 2200 °C [91]. Depending on the voltage applied, IR emitters are flexible in producing the wavelength required for a particular application.

### 4.2. IR Radiation and Heat Generation

IR radiation applied to biological materials is absorbed, reflected, or scattered in the matrix. To maximize the effectiveness of the heating process, the material to be exposed to IR radiation should have a high absorptivity. According to the wavelength range of the incident radiation, energy absorption mechanisms are classified as follows: (1) changes in the electronic state between 0.2 and 0.7 µm (ultraviolet and visible rays), (2) changes in the vibrational state between 2.5 and 1000 µm (FIR), and (3) changes in the rotational state greater than 1000 µm (microwaves) [89]. In general, biomolecules (in food) absorb IR energy most effectively through changes in their vibrational state, which may result in radiative heating [92]. Therefore, IR radiation induces changes in the electronic, vibrational, and rotational states of atoms and molecules in food, causing them to vibrate at frequencies of 60,000 Hz-150,000 Hz [87]. This vibration results in rapid internal heating and increases the water vapor pressure inside the tissues or food material [90].

When pulses are heated in an IR oven, the emitted radiation transmits through the air and is absorbed on the outer surface cell layers of the seeds, resulting in an excited, vibrational state of constituent molecules [93]. This generates heat that is conveyed into the surrounding tissues, partially cooking and drying the seed through increases in the internal temperature and the release of water vapor, respectively. As a result, pulse seeds treated with IR are partly dried and partially cooked. This has the potential to modify the physicochemical characteristics of macro- and micro-molecules and the functional properties they impart.

### 4.3. Effect of Seed Moisture Content (Tempering) on IR Heating

Tempering or adjusting seed moisture content (MC) is a common pre-treatment prior to IR heating. In this process step, water or an aqueous solution is applied to raise the MC to a predetermined level. The moisture level, time, and solution composition influence the quality of the end product and would allow the food processor to tailor the pulse ingredient to a specific application. Tempering is an important factor that determines the level of starch gelatinization, protein gelation, and cooking quality in IR heating [94]. According to Scanlon et al. [95], the superheated steam that is generated from seed moisture subject to high-temperature IR heating alters the physicochemical properties of starch and proteins in lentils. Moreover, chemical changes such as the Maillard reaction are also sensitive to MC and temperature conditions [95].

Some studies have shown that a higher seed MC prior to IR heating results in a higher degree of starch gelatinization [96]. An increase in the degree of starch gelatinization coupled with decreases in protein solubility was proposed to explain the accompanying increases in water absorption and cooking time observed at higher tempering moisture levels [19]. However, increasing the MC may also increase the amount of water that is evaporated during heat treatment [97]. Cenkowski and Sosulski [98] demonstrated the effect of MC on the cooking time of lentils. Tempered lentils (25.8 or 38.6% MC) IR heated to a final MC of 18% needed 15 and 10 min of cooking (boiling in water to reach the desired texture), respectively, against 30 min for the control without pre-heat treatment. According to the same study, IR heating gelatinized 45 to 65% of the starch in the lentil seed, and subsequent cooking of IR-treated seeds for 5 min gelatinized most of the remaining starch. To obtain the desired chemical and physical properties of pulses and pulse ingredients, the optimum moisture–temperature combinations need to be determined. For example, Pathiratne [17] reported a significant increase in the water-holding capacity (WHC) of flours obtained from lentil seeds tempered and IR heated at higher treatment levels (16 or 23% MC; 130, 150, and 165 °C) compared with lower moisture and temperature combinations (8%; 115 °C). 

### 4.4. Effects of IR Heating on Properties of Pulse-Derived Ingredients

The physicochemical and functional properties of pulse ingredients determine their potential applications in different food products. Nitrogen/protein solubility, WHC, oil absorption capacity (OAC), gelation, and emulsifying capacity (EC) are a few of the functional properties of pulses and pulse-derived ingredients that may be affected by IR heating. Table 4 summarizes the impact of IR heating of the pulses on the physicochemical or functional properties of different pulses. The ingredient processor has a lot of latitude to generate an IR-heated pulse ingredient to exhibit an appropriate balance of physicochemical and functional properties to best suit a particular food or meat application.

#### 4.4.1. Protein Solubility

Protein solubility is a critical factor that influences its functionality in different food products and can be measured as nitrogen solubility index (NSI) or protein dispersibility index (PDI). IR heating decreased the nitrogen solubility of lentil flour by 33–64% over a pH range of 2–9, which is suggested to be due to heat-induced denaturation of proteins that exposes the hydrophobic regions leading to their aggregation in aqueous solutions [103]. Tempered and heated legumes (8–10% MC; 140 °C) had reduced nitrogen solubility (pH 6.0) by 12–41%, while increasing IR-heating temperature and seed MC further decreased the nitrogen solubility [107]. The lower solubility of proteins could be detrimental to the functional properties of proteins, such as stabilizing foams, emulsions, or gels [108]. Therefore, the use of mild heating conditions can be suggested to achieve optimum functionality of pulse-derived ingredients.

#### 4.4.2. Starch Gelatinization

Starch granule gelatinization is the loss of molecular structure when exposed to heating under excess moisture conditions with accompanying viscosity increases and possible gel formation. During gelatinization, water is absorbed into starch granules, resulting in hydration, swelling, and ultimately the loss of birefringence and crystallinity [109]. Due to the fact that IR treatments involve tempering and heating, certain treatments may provide suitable conditions for varying degrees of starch gelatinization to occur. For example, 17–24% starch gelatinization was observed in tempered and IR-heated lentils (23% MC; 115, 130, 150, and 165 °C). However, no starch gelatinization occurred in lentils with less than 23% moisture heated to the same surface temperatures [17].

#### 4.4.3. Water-Holding Capacity (WHC)

WHC refers to the ability to absorb, retain, and physically entrap water. It is a critical functional attribute for food applications. Several studies have revealed that tempering followed by IR heating can increase the WHC of flour derived from pulses. According to Bai et al. [103], tempered and IR-heated chickpea flour (20% MC; 115 and 135 °C) were shown to have WHCs of 1.7–1.8 g/g, significantly greater than that of untreated flour (1.1 g/g). Another study reported similar effects for IR-heated lentil flours (16% or 23% MC; 150 and 165 °C), revealing that tempering both the moisture level and heating temperature have a significant impact on the WHC of the flour [17]. These effects are hypothesized to be due to the temperature-induced unraveling of the protein conformation, exposing an increased number of previously buried hydrophilic moieties, in addition to structural changes associated with increased starch gelatinization in which water is imbibed and vaporized, thus leaving greater porosity for water to adsorb or bind in the resulting flour [110]. In agreement, Bai et al. [103] observed a strong positive correlation (r = 0.83) between starch gelatinization and WHC for IR-heated (untempered or 20% MC; 115 and 135 °C) chickpea flours.

#### 4.4.4. Oil-Holding Capacity (OHC)

OHC is equally as important as WHC when it comes to the utilization of pulse ingredients in foods. Meat products may contain a high percentage of fat that needs to be retained within the product during processing and storage. The type of pulse, seed MC, and IR heating temperature affect the OHC of pulse ingredients. Flours with a higher concentration of hydrophobic amino acids are thought to have a higher oil-holding capacity. Pathiratne [17] indicated that high tempering moisture combined with IR heating at 115 and 130 °C increased oil absorption more (~75%) than that of flour from untreated seeds and seeds from the same tempering level heated to temperatures above 130 °C (~27%). It was also discovered that the OHC of lentil flour is inversely proportional to its WHC. The OHC of untreated chickpea flour was reported as 1.13 g oil/g, and IR heating (untempered or 20% MC; 115 and 135 °C) had no influence on the OHC of chickpea flours [103]. Similarly, Der [16] showed that tempered and IR-heated lentils from two different cultivars (15% MC; 135 °C) had no noticeable effect on the OHC. Although pulse proteins can play an important role in the retention of oil in emulsions, there is no evidence that IR heating affects the OHC of pulse proteins.

#### 4.4.5. Emulsifying Capacity (EC)

Emulsifiers provide a cohesive film over dispersed oil droplets in an aqueous medium, preventing structural changes such as coalescence, creaming, flocculation, or sedimentation. This property is important for ingredients added to meat products to stabilize emulsions and prevent finely chopped fat particles or liquid oil (dispersed phase) from coming together. Bai et al. [103] found that the emulsifying activity of tempered, IR-heated chickpea flour (20% MC; 135 °C) increased from 44 to 47% compared with non-tempered, non-heated flour. The increase in emulsion-forming characteristics is thought to be due to unraveling of the protein structure, exposing previously buried hydrophobic sites, effectively enhancing the oil-holding capacity of the flour. Emulsions are also thought to be stabilized by steric and electrostatic repulsive forces that push the two parts away from each other. These forces are created by the viscoelastic interfacial film and an increase in the viscosity of the continuous phase caused by starch gelatinization Bai and colleagues [103] found that the emulsifying capacity of chickpea flour was pos [111]. Itively correlated with gelatinized starch (r = 0.75, *p* < 0.01) and negatively correlated with protein solubility (r = 0.71, *p* < 0.01). Since IR heating and tempering can both improve starch gelatinization while decreasing protein solubility, they may have opposing effects on the ability of pulse flours to emulsify fat.

#### 4.4.6. Effects on Nutritional Properties

IR heating has been reported to impart minimal negative effects on the nutritional properties of pulses and may contribute to enhanced bioavailability of nutritional components in pulses, primarily through lowering enzyme inhibition and increasing the digestibility [16,17,18,94]. In most studies, IR heating did not significantly change the contents of carbohydrates, protein, and fat in pulses [16,17,18]. For example, Deepa [112] reported minimal changes in nutrient content as a result of IR heating, although partial gelatinization made starch easier to digest with improved sensory properties and shortened cooking time. In mung beans, IR treatment led to a significant retention of vitamins B1 and B3 [113]. IR-heated cowpeas (41% MC; surface temperature not reported) were successful in lowering trypsin inhibitor activity by 88–90% [114]. Zhang [115] showed IR heating to reduce the phytic acid content of beans, with greater reductions when the beans were soaked and cooked for longer time periods. 

#### 4.4.7. Effects on Enzymes

**Figure 3:**
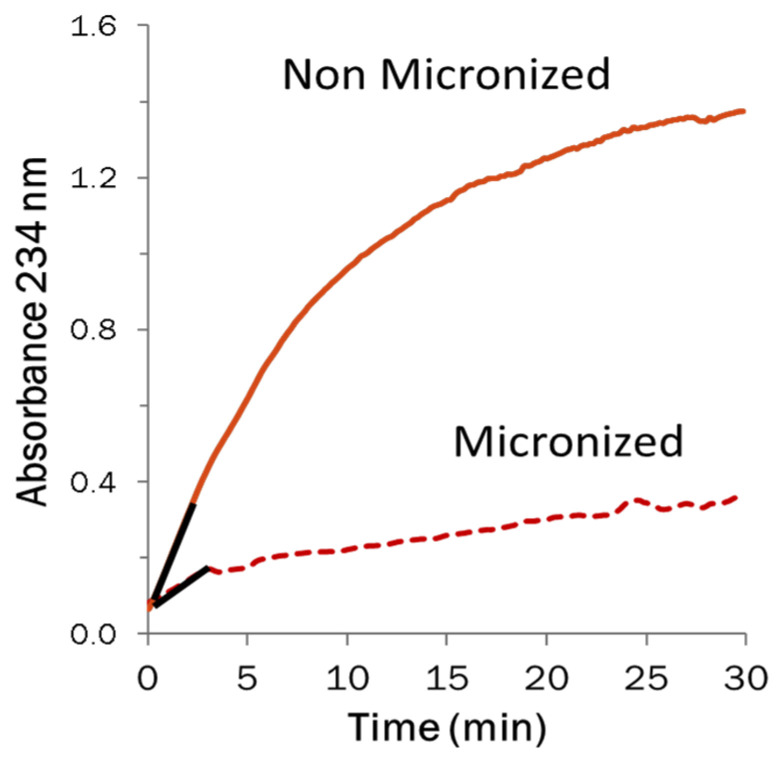
Effect of IR heating on lipoxygenase activity of lentil (one enzyme unit = Δ in abs of 0.001/min). (Reprinted with permission from Der [16]).

Increased seed temperatures (>100 °C) as a result of IR treatment may have an effect on enzyme activity by inducing conformational changes. Therefore, IR heating has been investigated as a method of inactivating oxidative enzymes, which are associated with the production of off-flavor and off-color in pulses. Shariati-Ievari and colleagues [116] examined the impact of IR heating (130 and 150 °C) on the lipoxygenase activity of chickpea and green lentil flours, where a significant decrease in lipoxygenase activity was observed. Another study found a 100-fold decrease in lipoxygenase activity in lentils that had been IR heated at 135 °C (Figure 3) [16]. Pathiratne [17] found that IR heating of lentil flours (115, 130, 150, and 165 °C) decreased lipoxygenase activity by 70–100%. In addition, the activity of peroxidase and trypsin inhibitors was reduced by 32–100% and 30–54%, respectively.

Results from Shariati-Ievari et al. [116] and Pathiratne [17] emphasize that increasing the temperature of IR treatment may boost the inactivation impact on enzymes. Lipoxygenase and peroxidase were inactivated by IR heat treatment of lentil seeds at 150 °C to negligible levels without affecting the activity of antioxidative enzyme superoxide dismutase [18]. These results show that IR heat treatment is an effective way to inactivate enzymes that are responsible in making pulses less acceptable. IR heating may be a preferred method of pre-treatment to enhance the vital functional and sensorial attributes of pulses before adding them into meat products.

#### 4.4.8. Effect on Lipids and Volatile Organic Compounds (VOC)

Polyunsaturated fatty acids such as omega-3 fatty acids are essential nutrients in the diet and are highly prone to oxidation. In pulses and meat products, VOCs are formed as oxidation products of unsaturated fatty acids. Lipoxygenases in pulses catalyze the oxidation of linoleic acid to generate VOCs. Shariati-Ievari et al. [116] investigated the effect of IR heating (130 and 150 °C) to determine its influence on volatile organic compounds (VOCs) of chickpea and green lentil flour. Heat-treated pulse flours had low lipoxygenase activity and low concentrations of several VOCs generated from polyunsaturated fatty acids (PUFAs). VOCs were successfully reduced by IR heating at 130 °C for chickpea flour; however, a higher heating temperature of 150 °C was more effective for green lentil flour. In chickpea flour, the concentrations of 1-pentanol, hexanal, 2-hexenal, hexanol, heptanal, furan-2-pentyl, and undecane were significantly decreased at 130 °C, with no effect on VOCs detected in green lentil flour. However, higher IR heating temperatures (150 °C) significantly decreased the levels of hexanal and 2-hexenal compared with the untreated lentil flour. These results indicate an influence of pulse type on the effectiveness of IR heating in the reduction of VOCs irrespective of their initial concentrations. In addition, this study reported that IR heating had little effect on the characteristic fatty acid content or composition of each flour but significantly increased omega-3 and n-6 fatty acids in lentil and chickpea flour treated at 150 °C in comparison with heating at 130 °C. Adding this flour to low-fat beef burgers increased their total omega-3 and -6 fatty acid content (Table 5). Chickpea flour naturally contains a higher level of omega-6 fatty acids than lentil flour (62% vs. 46%) [116]. IR heating showed a minor effect on the typical fatty acid content or composition of each flour but significantly increased omega-3 and -6 fatty acids in lentil and chickpea flour treated at 150 °C in comparison with flour treated at 130 °C. Based on these results, it is clear that IR heating is an effective method for controlling the production of VOCs, which are the primary cause of off or oxidized flavor of meat products (Table 5).

## 5. Performance of IR-Treated Pulses in Comminuted Meat Products

Ingredients derived from pulses retain nutritional and functional benefits of the carbohydrates, protein, micronutrients, and phytochemicals of the seed and can be employed in various meat product formulations. Raw/native pulse ingredients have been successfully incorporated as emulsifiers and binders in processed meat products [11,12,13,14,116,117,118]. However, limitations in terms of their effects on other product characteristics such as fresh meat color stability have been reported. For example, raw lentil flour was found to increase the oxidation of meat pigments, which was linked to the activity of the endogenous lipoxygenase enzyme [18]. Therefore, studies that can improve the useability of pulse flours in processed meat applications via pre-treatments such as IR heating have been carried out. This section of the review presents findings of studies in which IR-heated pulse ingredients were utilized in meat products. Emphasis was on IR-treated lentil flour in meat products as that was the focus of the majority of the studies available (Table 6). Parameters that are significant to meat product quality characteristics are mainly discussed. All studies discussed in this section have used a similar type of IR heating equipment (Figure 2) to process the seeds. The seeds have been heated to reach different surface temperatures as indicated in Table 6. The equipment consisted of a vibrating conveyor and a feeder that turned the seeds frequently as they passed beneath the IR emitters, providing uniform heating of the seeds.

### 5.1. Improved Retention of Redness in Uncooked Meat Products with IR Treatment

Color is an important consideration when purchasing uncooked burgers or fresh sausage and can be assessed using instrumental methods and visual observation. According to Fernández-Lopez et al. [119], the red color of beef burgers reduces within the first 5–6 days of storage. The CIELAB L*, a*, and b* values, which measure lightness, redness, and yellowness, respectively, are a very common and easy way to judge the color of meat. For example, the mean values of L*, a*, and b* in beef longissimus lumborum muscle at 24 h post-mortem were 37.6, 14.4, and 8.8, respectively [120].

**Figure 4:**
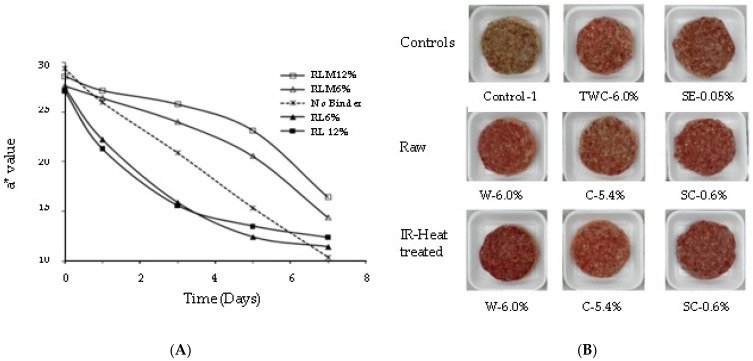
(**A**) Change in HunterLab a* values of raw beef burgers containing dehulled red lentil flour and stored at 4 °C from 0 to 7 days (RLM = red lentil IR-treated; RL = red lentil non-IR-treated). (Reprinted with permission from Der [16]). (**B**) Color of uncooked beef burgers formulated with green lentil cotyledon flour and hull fiber after a 12-week frozen storage at −18 °C (Control-1: no binder, TWC = toasted wheat crumb, SE = sodium erythrobate, W = whole seed flour, C = cotyledon flour, SC = hull fiber (Adapted with permission from Li [18]).

One of the most unique functionality benefits of incorporating lentil flour from IR-heated lentils was the improvement of color stability of fresh beef products during storage. Der [16] evaluated the color as the CIE color of uncooked beef burgers incorporating 6 and 12% (by weight) IR-treated dehulled lentil flour (15% MC: 135 °C) and non-IR-treated flour from dehulled green and red lentils as a binder to low-fat (10%) beef burgers stored at 4 °C (Figure 4A). Overall, redness (a*) decreased for all uncooked burgers during the 7 days of retail display (4 °C); however, the rate of redness reduction was slower when IR-treated lentil flour was incorporated. On day seven, the a* value of the uncooked burgers formulated with 12% IR-treated green lentils reached 17.4. The corresponding values of the burgers with non-IR-treated lentils (12%) and no-binder control were 11.04 and 10.4, respectively, showing a significant positive effect from IR treatment on red color retention. This prolonged redness retention after production could prove useful to decrease product loss at retail or at the consumer level. It is noteworthy that in the same study, a more rapid discoloration occurred with the addition of unheated lentil flour to low-fat beef burgers (Figure 4A). Other studies have also reported the improved color stability of uncooked low-fat beef burgers formulated with IR-treated lentil flour [18,118]. Burgers containing 6% IR-treated whole lentil flour had a significantly higher a* value (16.0) in comparison with the burgers with the same amount of untreated lentil flour (11.9) and no binder (9.7) formulations [117] on the fourth day in retail display at 4 °C. Similarly, Li [18] reported a lower magnitude of decrease in the a* value (10.7 vs. 15.8) from day 0 to day 3 (4 °C storage) in uncooked beef burgers formulated with 6% IR-treated and untreated whole lentil flour. These findings confirmed that formulating uncooked meat products with IR-treated lentil flour rather than untreated lentil flour would be especially important for extending raw color stability.

One study set out to define the optimum IR heating conditions of green lentils for maintaining meat product redness [117]. Heating temperatures of 115 and 135 °C outperformed temperatures of 150 and 160 °C in terms of maintaining product redness (a*) stability over a 4-day period in a retail display when these flours were combined with uncooked, low-fat beef burgers [117]. Based on the characteristics of lentil flour, the author concluded that 135 °C, with 23% seed MC, was the optimal heating temperature for IR pre-treatment.

The pulse type, market class, variety, and quantity applied of IR-treated pulse ingredients used in meat products may also influence color stability. However, according to Der [16], both the inclusion level of dehulled lentil flour (6% vs. 12%) and the market class of the lentil (green vs. red) had no significant impact on the a* value during the 7 days of storage, irrespective of the IR treatment. This suggests that a 6% difference in the addition level between two cultivars of the same pulse may have no effect on their functionality in meat products when using the dehulled seed flour. However, there may be a greater effect on color stability when the whole seed flour or seed coat is used. Different market classes of pulses, including lentils, have significantly different seed coats in terms of bioactive/antioxidant compounds [57,58,121]. However, only limited data is available on the effect of IR treatment on seed coat properties with respect to their functionality in meat products.

More recently, focus shifted to investigating which part of the lentil seed could be attributed to color stability in meat products. Li [18] investigated the effect of IR treatment on whole green lentil flour (at an inclusion level of 6.0% by weight) and its separated seed components, cotyledon flour (at 5.4%), and hull fiber powder (at 0.6%) as binders in beef burger formulations. The levels of dehulled lentil flour and hull fiber represented their respective proportions in the whole seed flour. According to the findings of this study (Figure 4B; all burgers made with IR-treated lentil components had higher a* values (>19.5) than the respective control formulations made with non-IR-treated ones (<18.1)). It was interesting to note that the ability of hull fiber to maintain the color was similar to that of sodium erythorbate, which is a common antioxidant used in meat products. The effect of IR-treated lentil (23% MC; 150 °C) ingredients was associated with completely deactivated oxidative enzymes while maintaining antioxidant enzyme activity due to heating [18]. Therefore, when lentil flour is added to fresh, ground meat products, IR pre-treatment is recommended for whole and cotyledon flours to reduce the activity of oxidative enzymes. Binder (IR-treated) concentrations of 6% were found to be superior for providing color-preserving effects compared to those of commercial binders such as toasted wheat crumb [16,18] and wheat flour [16]. The addition of whole lentils or hull fiber may provide additional color-preserving effects due to higher levels of antioxidant compounds, particularly phenolic compounds, than those found in dehulled flours [18,58], although this may depend on the severity of the dehulling process, as typically not all hull is removed commercially.

### 5.2. Decreased Metmyoglobin Formation during Uncooked Meat Product Storage with IR Treatment

Myoglobin (Mb) is the main meat pigment that determines the red color of meat. Therefore, maintaining the brown-colored metmyoglobin (metMB), the oxidized form of Mb, at low levels is vital for meat products. Section 5.1 described that IR-treated lentil seed ingredients can extend the color stability of uncooked beef burgers over a 7-day retail display period at 4 °C, which could be explained by the low level of metMb in the products. Investigating the redox state of Mb, Li [18] reported a strong negative correlation between the a* value and the metmyoglobin level, confirming that metMb accumulation is the cause of the decrease in the redness of beef burgers. Burgers containing IR-treated lentil seed components had lower metMb levels. According to Li [18], the concentrations of metMb in low-fat beef burgers with the addition of 6% whole seed flour, 5.4% dehulled seed flour, and 0.6% hull fiber, respectively, were 38%, 41%, and 36%, as compared with no binder controls (55–59%) and burgers with 6% untreated whole seed flour (70%). The higher accumulation of metMb corresponded to lower a* values in the respective products. This study emphasized the importance of IR pre-treatment by demonstrating that the accumulation of metMb in uncooked products formulated with untreated lentil was higher than in products with treated lentils and even higher than in no-binder controls.

There was consensus that the delay in the oxidation of Mb and the stability of the red color in products formulated with heat-treated lentils was related to the inactivation of oxidative enzymes (such as lipoxygenase) and the concomitant increase in antioxidant activity [16,17,18]. As a result of the IR heat treatment, lipoxygenase activity and, therefore, free radical formation would be reduced. In addition, high-temperature conditions during IR heating may also result in Maillard browning reactions, yielding compounds with antioxidant properties. This was also shown by Acar et al. [122], who related the increase in antioxidant activity in pulses to high-temperature processing techniques such as roasting. In addition, Mb is thought to be protected from oxidation by the antioxidant phenolics in lentil seed components and the increased ferrous ion chelating ability of soluble protein released by heat treatment [18]. Indeed, raw (unheated) lentil flour had no ferrous ion chelating ability, while flours from IR-treated seeds showed increases of 36 to 39% over baseline values. Allen and Cornforth [123] found that metal chelators are more effective than radical scavengers at preventing the oxidation of oxymyoglobin to metmyoglobin. Thus, metal chelation ability of heat-treated pulse flours may be an important factor in increasing the color stability of Mb and could be a functional property to be measured more often in ingredient evaluations. Further investigations are needed to confirm improvements in color stability with the addition of IR-treated lentils to meats of other species, but it is speculated that this effect may only be observed in meats with sufficient myoglobin content as no effect on color stability was observed during the retail display of mechanically separated chicken meat patties with 6% commercial IR-treated lentil flour [81].

### 5.3. No Change in Color Stability with IR Treatment in Uncooked Frozen Meat Products

Frozen meat products also benefit from formulating with pulse ingredients. An assessment of lentil ingredients in frozen beef burgers [18] confirmed that the color stability can be extended in uncooked frozen burgers. However, unlike the refrigerated storage, the IR treatment had no beneficial effects on color stability beyond that shown with the addition of untreated lentil ingredients. When compared with the no-binder meat control, adding lentil flour from either IR-treated or untreated seeds to beef burgers resulted in higher a* values (>20) and lower metMb concentrations after 6 to 12 weeks of frozen storage. It was hypothesized that the frozen storage conditions delayed any pro-oxidant activity of the lipoxygenase in the untreated lentil flour. In contrast, the addition of sodium erythorbate at 0.05% [18] caused the surface color of frozen beef burgers to be dark purple due to the high level (>45%) of deoxymyoglobin (deoxyMb) that formed from the beginning and throughout the entire storage period (12 weeks). This effect was not found in the products with lentil components, which showed enhanced redness throughout the frozen storage period, also in contrast to the darkening observed for the no-binder control burgers.

Similar to the results observed for refrigerated burgers, IR-treated lentil flour exhibited stronger antioxidant effects in beef burgers than toasted wheat crumb (6%), due to the endogenous antioxidants, phenolic compounds, and enzymes. Overall, work by Li [18] suggested that adding lentil flour that has been IR-heated can help beef burgers retain their red color when they are stored at either refrigerated or frozen temperatures by acting as an antioxidant. This means that these products can stay desirable for a longer time at retail or in the freezer, cutting down on food wastage. A particular benefit of lentil flour addition prior to the frozen storage of beef burgers is that it allows commercial manufacturers to prepare in advance of the busy summer burger season. Nonetheless, this is the only study conducted to date assessing the impact of IR-treated pulses in frozen meat products, requiring the attention of researchers in the future to confirm these findings in other meat products. 

### 5.4. Decreased Lipid Oxidation during Refrigerated or Frozen Storage of Meat Products Due to IR Treatment

Oxidation of unsaturated lipids is a major contributor to the loss of quality in both fresh and processed meat, which continues during refrigerated or frozen storage of the products. The quality of oxidized meat degrades in different ways, including changes in color, flavor, texture, and nutritional value. There is clear evidence that pulse ingredients that have been subjected to IR radiation minimize lipid oxidation in both fresh and frozen meat [17,18]. In low-fat beef burgers, IR-treated dehulled lentil flour (6 and 12%) delayed lipid oxidation measured in terms of thiobarbituric acid reactive substances (TBARS) during frozen (−20 °C) storage [16]. The author reported that low-fat burgers containing IR-heated lentil flour had TBARS levels between 0.6 and 0.8 mg/kg, but untreated lentil flour burgers had TBARS levels between 1.7 and 1.9 mg malondialdehyde/kg after 11 weeks of frozen storage. In addition, there was no statistically significant difference between the levels of flour addition and the TBARS values, showing that 6% IR-treated lentil flour is sufficient to stabilize meat lipids. Pathiratne [117] observed that whole lentil flour prepared from IR-treated seeds decreased the oxidation of meat lipids in uncooked beef burgers stored at refrigerator temperature. Unsaturated lipids were shown to be stable (1.8 vs. 0.9 mg MDA/kg) after four days of refrigerated retail display when IR-treated seed flour was added.

Optimum reductions in the lipid oxidation of beef patties with the addition of IR-heated lentil flour appear to be related to the use of higher-temperature IR heating conditions. IR treatment at 130 °C and 150 °C was more effective than 115 °C in delaying oxidation, and this was attributed to the lower (or lack of) residual activity of the oxidative enzymes [117]. It has been established that the activity of lipoxygenase enzymes reduces as the IR heating temperature increases. After being subjected to infrared treatment at temperatures ranging from 110 to 140 °C, the level of lipoxygenase activity in black beans decreased from 30 × 10^5^ units/mL to 1.3 × 10^5^ units/mL [124]. With regard to lentils, the lipoxygenase activity dropped from 134.6 × 10^5^ units/g protein to 40.5 × 10^5^ and 0.4 × 10^5^ units per g at heating temperatures of 115 °C and 130 °C, respectively, and there was no significant difference in lipoxygenase activity when the lentil was heated above 130 °C [117]. Additionally, the author found that the heat-induced suppression of lipoxygenase was not affected by seed tempering MC. Thus, it appears that lipoxygenase activity in pulses can be suppressed by heating them to temperatures above 130 °C using infrared radiation. For routine quality assurance purposes, the lack of lipoxygenase activity can be determined with a simple colorimetric test [125] rather than the more quantitative assays performed in the studies above.

The antioxidant properties of the different types of lentil ingredients were found to be at their highest in whole lentil flour and hull fiber derived from heat-treated seeds, which is in agreement with effects previously described for color retention [18]. Low-fat beef burgers made with IR-treated whole lentil flour had lower TBARS (1.25 mg MDA/kg sample) than those made with dehulled seed flour (2.68 mg MDA/kg sample), illustrating the beneficial contribution of hull fiber to oxidation reduction. Although the weight proportion of the hull is much lower (8.2–11.4% of seed weight), catechins, procyanidins, flavanols, and flavones are concentrated in the hull and the main contributors to the total phenolic content of lentils, whereas cotyledons (dehulled flour) only provide a small proportion of phenolic compounds, mainly cinnamic and benzoic compounds [126]. This abundance of antioxidants correlates with the low TBARS. On the other hand, the burgers made with untreated dehulled seed flour had TBARS values of 4.63 mg MDA/kg sample, which further demonstrates the benefit of employing IR pre-treatment of pulses. 

The susceptibility of meat products to lipid oxidation is influenced by a variety of variables, including product type, meat species, fat composition, and other additives. Even though there have been few research studies conducted on various meat products, the available evidence indicates that meat products benefit from the application of IR pre-treatment to pulses when they are utilized as an ingredient in the formulation. Burgers are formed meat products made of ground meat mixed with other ingredients and additives, and they may contain varying amounts of fat. In low-fat (<10% fat) beef burgers, IR-treated lentil ingredients have increased the stability of the unsaturated lipids [16,17,18]. When lentil whole seed flour was added at a 6% level, the TBARS were maintained below 2 mg MDA/kg in low-fat beef burgers stored refrigerated for up to a week or frozen for up to 12 weeks, whereas the corresponding values for the burgers formulated with the untreated flour were as high as 4 mg MDA/kg [16,17,18]. This suggests that the addition of IR-treated pulses to low-fat meat products will be useful to extend their oxidative stability.

Thus far, most studies on the addition of IR-heated lentil flour focused on low-fat meat products. Recently, Kim and Shand [119] studied the effect of IR-treated lentils in bologna prepared with pork shoulder muscles, mechanically separated pork, and back fat. The fat content of this bologna was between 15 and 18%. They observed that the TBARS of bologna products with 6% commercial IR heat-treated flour (NutraReady™) was 0.30 mg MDA/kg after 12 weeks of storage at 4 °C. In this study, there was no control formulation with untreated lentil flour; however, they compared the antioxidant efficacy of the lentil flour against other antioxidant ingredients, sodium nitrite, and beet powder. Interestingly, there was no significant difference in the TBARS levels among the products (TBARS between 0.23 and 0.30 mg MDA/kg), indicating the potential of replacing other antioxidant ingredients with IR-treated lentils. Similarly, Pathiraja [81] found that the antioxidant capacity of IR-treated whole lentil flour (NutraReady™) was comparable to that of nitrite (125 ppm) and sodium tripolyphosphate (0.5%) when used in mechanically separated chicken wiener sausages and bologna, respectively. Further studies with other higher-fat meat products are needed to confirm similar efficacy in those products.

**Figure 5:**
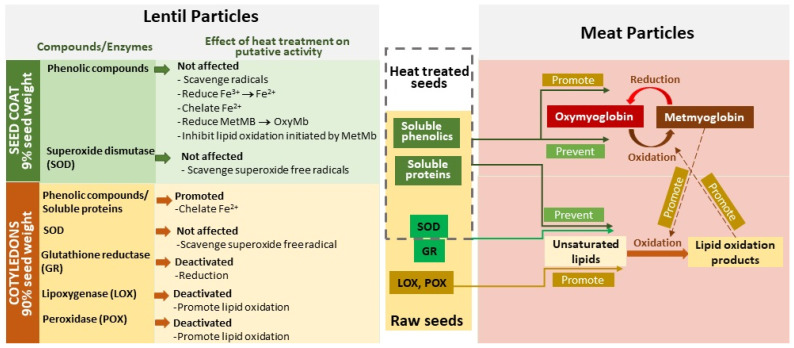
A summary of observed (and postulated) effects of heat treatment (**left**) on phenolic compounds and antioxidative and oxidative enzymes within the lentil seed coat (hull fiber) and cotyledon (dehulled seed flour) separately and their effects on the oxidation of oxymyoglobin and unsaturated lipids in a minced meat particle (**right**). (Adapted with permission from Li [17]).

The oxidation of myoglobin and unsaturated lipids in muscle-based foods have a close relationship, and some studies show inter-dependencies [127]. The studies by Der [16], Pathiratne [117], and Li [18] clearly showed pro-oxidant and antioxidant activity of pulse-based binders on the oxidation of meat color pigments and unsaturated lipids. The plausible explanation for the improvement of pulse ingredients by IR heat treatment is the inactivation of lipoxygenase due to the seed temperature increase and steam generated from moisture within the pulse seed. Studies have shown there is an optimum seed moisture level of 22–23% [17,106] and temperature >130 °C [17,106,125] that is effective in inactivating lipoxygenase. Li [18] further determined that the maintenance of a reducing environment and the activity of antioxidant enzymes and compounds are required for maintaining the balance between oxy- and met myoglobin conversion in fresh meat particles of burgers; therefore, the added binder ingredient can alter the equilibrium depending on the nature of the activity of its constituents. Figure 5 depicts a simplified mechanism for the intervention of lentil binders (with and without heat treatment) in this complex cascade of biochemical changes occurring in fresh comminuted meat particles.

### 5.5. IR-Heated Pulse Flours Show Effective Liquid-Holding Properties as a Binder in Meat Products

Emulsion-type meat products (described in Section 2) consist of a multi-phasic matrix where water and fat globules are immobilized and physically entrapped. Thus, the binding properties of the added ingredients are critical for maintaining the integrity of the emulsion system during thermal processing and storage. Using pulses in meat products has been shown to improve the water- and oil-holding properties of the meat products [11,12,13,14,116,118]. Assessing the cook loss (the weight loss during cooking) and expressible moisture (the weight of liquid loss due to centrifugal force), Unatrakarn [106] concluded that the IR treatment of chickpeas (6% level) has no detrimental effect on their liquid (water and fat) holding properties in cooked low-fat pork bologna. They found that the cook loss, expressible moisture, and purge loss (the liquid loss due to gravity during storage) of bologna containing IR-treated chickpeas were not significantly different from those formulated with untreated flour, with values ranging between 0.32 and 0.43%, 14 and 16%, and 3 and 5%, respectively. Additionally, they reported that neither the IR heating temperature nor the seed tempering moisture had any impact on the liquid-binding properties in the meat products. In terms of formed meat products, Der [16] and Nicholson [124] also reported comparable results with IR-heated lentils (heated to 135 °C) and black beans (heated to 100, 110, and 120 °C), respectively, added to low-fat beef burgers. Both studies concluded that IR heating in the studied temperature regimes had no detrimental effect on the lentil or black bean flours with respect to the liquid-binding properties of the burgers. Shariati-Ievari [116] reported no effects on the liquid-binding properties; the drip loss and shrinkage of both the IR-treated (130 and 150 °C) chickpea and lentil flour incorporated (at a 6% level) into beef burgers showed no significant differences between pulse types. All these studies showed no additional ability of the IR treatment to improve the functionality of these pulse ingredients with respect to liquid holding in meat products. This is somewhat surprising as IR treatment did change the flour properties when the dry ingredients were tested alone (Section 4.4.3 and Section 4.4.4). Perhaps the addition level of the flours was too low to show any subtle changes in the meat matrix properties.

Several studies have compared the liquid-binding properties of IR-treated pulses in meat products against commercial binders. For example, Der [16] and Nicholson [124] compared the liquid-binding properties of low-fat beef burgers formulated with IR-treated lentil and black bean flour, respectively, against toasted wheat crumb and regular wheat flour and found that the binding properties were similar in all products. The use of toasted wheat crumbs or regular wheat flour as a binder in ground meat products is well established by the meat industry and accepted by consumers. However, lentil-flour-containing products can cater to consumers with celiac disease or other wheat intolerances. As such, several studies [16,18,118] demonstrated an extra benefit to lentil flour, i.e., the ability to retard the oxidation of Mb in uncooked meat over the traditional binder, particularly with improvements achieved by IR heat treatment. 

Alkaline phosphates are commonly used to improve the water retention and textural properties of cooked meat products by increasing the pH and ionic strength [85,128,129]. With the increasing consumer demand for clean-label products, the replacement of chemical ingredients such as phosphates is a consideration by the meat industry. Pathiraja [81], investigating the use of commercially available IR-heated whole seed lentil flour (NutraReady^®^) as a replacement for phosphates (sodium tripolyphosphate: STTP) in uncured bologna-type chicken sausage, confirmed that lentil flour (6%) was effective in delivering the liquid-binding properties similar to synthetic phosphates.

### 5.6. Maintenance of Textural Properties in Meat Products with IR Treatment

**Figure 6:**
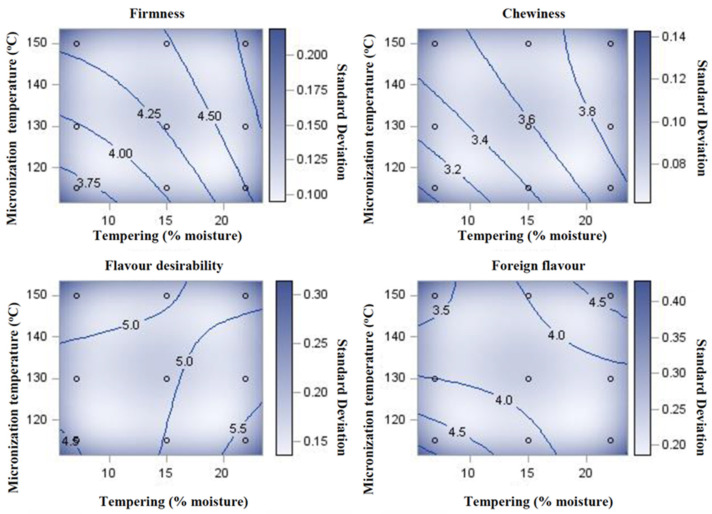
Contour plot for sensory firmness, chewiness, flavor desirability, and foreign flavor in bologna with added chickpea flour. Firmness: 5 = slightly firm, 4 = slightly soft, and 3 = moderately soft; chewiness: 4 = slightly easy to chew, 3 = moderately easy to chew; flavor desirability: 6 = moderately desirable, 5 = slightly desirable, and 4 = slightly undesirable; foreign flavor: 5 = slightly intense, 4 = slightly weak, and 3 = moderately weak. (Reprinted with permission from Unatrakarn [106]).

Texture is a multi-parameter sensory property that is linked to the product’s structure, mechanical, and surface properties [130]. Proteins are the primary structural and functional constituents of processed meats. Additionally, Chen et al. [131] and Carballo et al. [132] reported that the inclusion of starch in heat-induced meat emulsions creates a more compact protein matrix. Although the addition of pulse ingredients has had varying effects on the textural properties of meat products [11,12,13,14], IR treatment has a minimal impact on the texture-forming properties of pulse ingredients beyond that of the comparable unheated flour (Table 6). The texture profile of meat products expressed in terms of hardness (N), chewiness (N*mm), springiness (mm), fracturability (N), and cohesiveness in all studies has been carried out using a texture analyzer. Agreeing with that, the shear force (29–38 N), hardness (69–112 N), cohesiveness (0.32–0.44), and springiness (64–65%) of low-fat beef burgers were not affected by the IR treatment at 135 °C [16]. The findings of another study [106] assessing the same effects of IR-treated chickpea flour (6% addition level) in pork bologna were generally consistent with those findings of Der [16]. However, Figure 6 shows that the sensory firmness and chewiness of pork bologna with added chickpea flour were increased at higher IR heating temperatures in combination with higher levels of tempering, which indicated that beneficial effects on the texture of meat products with IR heating of pulses may be subtle but may exist. One other important finding was that the texture of bologna made with IR-heated chickpea flour was the same as that of bologna made with regular wheat flour, confirming the opportunities for pulse-based binders in developing processed meat products for ingredient origin-sensitive consumer groups. Moreover, Pathiraja [81] also demonstrated that the IR-heated lentil flour had the same functionality as commercial binders such as isolated soy protein and corn starch in modifying the texture of bologna and wiener sausages formulated with mechanically separated chicken. 

The type/market class and quantity of the pulse used, as well as the type of product, will determine the overall effect on their texture. The evidence available to date, however, shows that the type of pulse (chickpea, lentil, or black bean) or the level of the addition of IR-heated pulse flours has little impact on the texture properties beyond that of the untreated flour control. For example, the application of IR-treated lentil flour at 6% to 12% in burgers [16] produced texture properties similar to their untreated control products. However, no studies are available that examined high levels of the addition of IR-treated pulse flours to meat products.

### 5.7. Consumer Acceptability of Meat Products with IR-Treated Pulses Generally Positive

The use of pulse ingredients influences the sensorial quality of meat products [12,14,16,53,79,119,133,134] and thus their consumer acceptability. Studies on IR-treated pulses showed a decrease in pulse flavor [124] and less VOC [116], and this has translated into less pulse flavor when incorporated in meat products [16]. Several studies have confirmed that the overall flavor acceptability was increased in beef burgers that incorporated IR-treated pulses over untreated pulses [16,116], with a reduction in off-flavor [16]. In another study, however, trained panelists noticed a foreign flavor described as beany, bitter, or nutty in non-cured chicken sausages made with commercially available IR-heated whole seed lentil flour (NutraReady^®^, 43% starch and 25% protein), despite the fact that the product was IR treated [81]. It was found that increasing the concentration of lentil flour from 2 to 8% intensified the foreign flavor and aftertaste in chicken wiener sausages but did not change the overall acceptability, and the consumer panelists were unable to recognize the difference in lentil flour concentration. Further, the flavor properties of meat products formulated with IR-treated lentil flour were similar to those of binders such as isolated soy protein and modified corn starch [81] and toasted wheat crumb and wheat flour [16].

**Figure 7:**
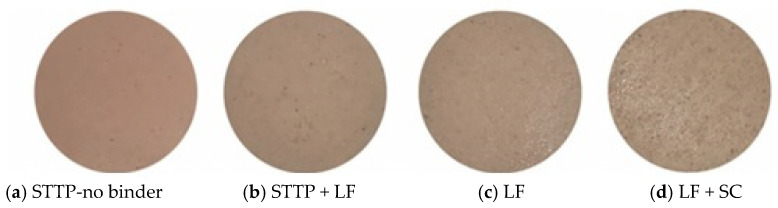
Cross-sectional view of cooked chicken bologna. (**a**) STPP-no binder: 0.3% sodium tripolyphosphate only; (**b**) STPP+LF: 0.3% sodium tripolyphosphate +6% lentil flour; (**c**) LF: 6% lentil flour; and (**d**) LF+SC: 6% lentil flour + 0.6% seed coat. (Reprinted with permission from Pathiraja [81]).

The appearance/color of meat products is another sensory property that consumers critically consider. Pulses, especially those with colored seed coats, contribute to the product color and may be difficult to mask in meat products. In chicken bologna, the addition of IR-treated lentil flour and seed coat (Figure 7b,d, respectively) changed the visually observed color of the product, and the instrumental color measurement also confirmed that the samples treated with seed components had lower L* values and were darker than control samples with no binders (Figure 7a).

This effect was also reflected by the redness (a*) values; a lowering of the red color was observed when lentil components were added to bologna, and a higher redness was observed in the control sample. Pulse ingredients can bring a grainy appearance when seed coat component-rich material is used as observed by Xu [53] with pork bologna products containing lentil bran. A similar effect was reported for chicken bologna formulated with IR-treated lentil whole flour [81]. However, Sharaiti-Ievari et al. [116] found that IR treatment had a favorable effect on lentils and chickpeas. When IR-treated pulses are added to beef burgers, the appearance and overall acceptability improved when compared with those with added untreated pulse flours. Seed processing needs to be optimized for IR temperature and degree of tempering to deliver the optimum sensory properties in the final product and to ensure the longest storage life. Pulses that are IR-pre-treated provide alternatives to the binders in the market and can improve the final product. Promisingly, the appearance, flavor, and texture acceptability of meat products formulated with IR-treated lentil flour were comparable to those formulated with commercial binders [16,81], showing the opportunity for their utilization at the commercial level. In addition, these show superior antioxidant properties and unique color stabilizing effects in raw beef products. Although the available studies are limited to the effects of IR-heated pulse ingredients in certain meat products, the findings are promising for the applicability of IR technology for improving pulse-based ingredients, particularly pulse flours in meat products.

### 5.8. Overall Benefits and Limitations of Using Infrared-Treated Lentil Ingredients in Processed Meat Products

Today’s food industry is reengineering processed food formulations that can deliver sufficient dietary proteins, consisting of both animal and protein sources. Blending meat and plant-based ingredients is an ideal approach to reduce meat content and incorporate plant proteins and other nutrients, as well as bioactive phytochemicals, into products without compromising the overall nutritional and sensory quality. In general, pulses, including lentils can be blended with meat and bring comparable nutritional profile to that of animal muscles. Lentils, in particular, can be processed to yield several ingredients differing in nutrient/chemical composition and techno-functional characteristics, as illustrated in Figure 1. Lentil ingredients would provide almost all the benefits listed in Table 4 to meat products. Although lentil ingredients function as a suitable binder for meat products, there are some limitations affecting their broader use, such as the presence of prooxidants, mainly oxidative enzymes, as well as nutritionally undesirable compounds. IR heating is an effective pre-treatment for controlling or overcoming these limitations and enhancing the functionality performance of lentil flour ingredients in meat products (Table 6).

The enhancement of the antioxidant properties of lentil ingredients was one of the most distinctive functional benefits of IR heating of lentils. This particular improvement resulted in extending the stability of the color and delaying lipid oxidation in both raw and cooked meat products, in either refrigerated or frozen conditions. An increase in oxidative stability in combination with the red color retention (less brown color) of meat products reduces the rejection of meat products at the grocery aisle due to low consumer appeal. The incorporation of lentil flour into meat products increased the liquid-binding capacity and thus the product cook yield as sufficiently as its untreated counterpart. The product cook yield is a significant quality consideration of the cooked, ready-to-eat product offerings at home and at food services. The effect of IR treatment on the textural properties and nutritional composition was minimal, having no detrimental effects compared with the commercial binders used in meat products. IR heat-treated lentil flour is a natural ingredient possessing antioxidant properties that can be used in developing clean-label products. The limitations of lentil flour, especially whole flour, could be the contribution to foreign flavor and, at high addition levels, the masking of the reddish color of the products. Although IR pre-treatment did not show a significant control to these limitations, these effects depend on the level of addition to the product, necessitating studies to optimize the level of addition in meat products without compromising product flavor and taste.

Overall, adding IR-treated lentil ingredients promotes meat product yields, oxidative stability, nutritional availability, and sustainability measures associated with animal foods. In the development of lentil-enriched meat products, the use of IR heat treatment widens the applications beyond the primary functions of binders. Introducing this ingredient into plant–meat blended products could bring viability and improve the sustainability of protein-rich food products.

## 6. Overall Summary

The macro-components of pulses provide the necessary binding ability of meat particles in comminuted meat products, stabilization of heat-treated meat-fat emulsions, and absorbent function of expressed liquid from meat particles during heat treatment. Heat pre-treatments, particularly those that use IR heat, have been effective in modifying macromolecules and minor components of pulses, as evident from the research carried out with lentils. The changes brought about by IR pre-treatments have been beneficial to meat products. The prolongation of a fresh red color, lowering of lipid oxidation products, and flavor changes were the most prominent benefits of IR pre-treatment, while the texture-forming properties were generally not affected significantly from those observed with the addition of raw, unheated pulse ingredients. IR-treated pulse ingredients, especially lentil-based, may also be used to replace commercial binders such as toasted wheat crumbs, isolated soy protein, and corn starch, as well as synthetic antioxidant compounds, in the transition to clean-label products. Thus, in the development of pulse-enriched meat products, the use of IR heat treatment further widens the applications beyond the primary functions of binders and will be a viable approach toward improving the sustainability of protein-rich food products. The effects of IR heat treatment on the properties of pulses and how they perform in meat products, however, were mostly limited to lentils and chickpeas, especially with whole and dehulled flours in a limited number of meat products. Studies carried out so far show the effects of IR treatment vary depending on the chemical composition of the seed components, seed coats and cotyledons, and thus how they affect the components of meat in product systems. Future research needs to focus on assessing the effects of IR heating on different pulses and their derived ingredients and their impact on a wide range of meat products. The efforts to date have been successful through the cooperation of scientists across a number of disciplines. Expanding on the multi-functional benefits of pulses requires an intimate knowledge of the ingredient functionalities and requirements of the specific meat system.

## Figures and Tables

**Table 1:** Standards for specific edible meat products in Canada.

Meat Product	Mandatory Ingredients	Compositional Requirements
Meatballs (uncooked), meat burger (uncooked)	Fresh boneless meat or fresh mechanically separated meat or both	Min. 11.5% meat product protein, min. 13% total protein
Meatballs (cooked), meat burger (cooked)	Boneless meat or mechanically separated meat or both	Min. 9.5% meat product protein, min. 11% total protein
Sausage, salami, wiener, frankfurter, bologna, pepperoni	Boneless meat or meat by-product or mechanically separated meat, or any combination of them, preservative	Min. 9.5% meat product protein, min. 11% total protein

Source: Canadian Food Inspection Agency [27].

**Table 2:** Nutritional composition of dry peas, lentils, and chickpeas (value per 100 g of whole seed flour).

Nutrient	Peas	Lentils	Chickpeas
Carbohydrates (g)	61.6	63.4	63
Dietary fiber (g)	22.2	10.7	12.2
Total sugars (g)	3.14	2.03	10.7
Protein (g)	23.1	24.6	20.5
Fat (g)	3.89	1.06	6.04
Calcium (mg)	46	35	57
Iron (mg)	4.73	6.51	4.31
Potassium (mg)	852	677	718
Zinc (mg)	3.49	3.27	2.76
Vitamin A (µg)	7	2	3
Vitamin C (mg)	1.8	4.5	4
Thiamin (mg)	0.719	0.87	0.47
Riboflavin (mg)	0.244	0.21	0.21
Niacin (mg)	3.61	2.60	1.54
Vitamin B-6 (mg)	0.14	0.54	0.53
Folate (µg)	15	479	577

Source: USDA [32].

**Table 3:** Potential advantages and disadvantages of adding pulses to meat products.

Property or Function of Pulses	Potential Effect on Meat Products
Advantages Water and oil retention properties	Improved juiciness, moist mouthfeel, flavor, and product yield, provide firmer texture
Contribution of protein	Increase protein content
Contribution of fiber	Increase dietary fiber content
Contribution of antioxidant compounds (ex: phenolic compounds, antioxidative enzymes etc.)	Increase oxidative stability of lipids, protein, and pigments, improve color stability, increase bioactivity
Disadvantages	
Contribution of pro-oxidative compound (ex: oxidative enzymes)	Increase lipid, protein, and pigment oxidation, reduce color acceptability, development of off-flavor
Contribution of color	Reduce redness of fresh meat products, increase dark color of cooked meat products
Contribution of antinutritive factors	Affects availability and digestibility of proteins and minerals
Contribution of fiber	Products become softer and less juicy

**Table 4:** Effects of infrared heating of pulses on their physicochemical or functional properties.

Pulse (Cultivar)	Infrared Heating Temperature (Seed Temperature) Seed Moisture Content after Tempering	Effects on Physicochemical or Functional Properties	Reference
Pea seeds (Pomorska)	180 °C	Decreased compressive resistance Shortened cooking time	[99]
Cowpea (Blackeye, Bechuana white, Glenda, Dr. Saunders)	153 °C 41%	Reduced cooking time by 28–49% Increased roasted aroma and flavor Mushy texture and splitting	[100]
Lentils (Laird)	138 and 170 °C 33%	Decreased soluble protein, phytic acid, and neutral detergent fiber Increased gelatinized starch and pectic substances	[96]
Cowpea, pea, and kidney bean	90 °C	Decreased tannins, phytic acid, trypsin inhibiting activity, and oligosaccharides content	[101]
Lentil (CDC Greenstar, CDC Invincible, CDC Maxi)	130 and 150 °C 24%	Decreased average particle size Increased water-holding, gelatinized starch, protein denaturation, and breakdown of amylose	[102]
Chickpea (Desi)	115 and 135 °C. 20%	Decreased protein solubility No effect on emulsion and foaming stability, OAC Increased WHC	[103]
Yellow peas	140 °C	Increased dry matter digestibility and digestible protein content	[104]
Lentil (Laird and Eston)	120 °C 20, 30, and 40%	Decreased protein solubility, cooking time Increased gelatinized starch	[94]
Yellow pea	110–115 °C 16%	Reduced protein solubility, foaming capacity Increased WHC and OAC	[105]
Lentil (Red and green)	135 °C 15%	Reduced lipoxygenase activity No effect on OAC Increased WHC	[16]
Chickpea	115, 130, 150, and 165 °C	Reduced lipoxygenase activity Increased starch gelatinization, OAC, and WHC	[106]
Lentil (Eston)	115, 130, 150, and 165 °C 16% and 23%	Decreased protein solubility Reduced lipoxygenase and peroxidase activity and trypsin inhibitory activity	[17]
Lentil (CDC Maxim, CDC Greenland)	115 and 150 °C 23%	Decreased lipoxygenase, peroxidase, and glutathione reductase activity No effect on superoxide dismutase Increased Fe^2+^ chelating activity	[18]

**Table 5:** Fatty acid composition (% of total fatty acid) of raw low-fat beef burgers containing 6% of infrared (IR)-heated and unheated chickpea and lentil flour.

Fatty Acid	Control (No Binder)	Chickpea Flour (6%)			Lentil Flour (6%)		
		Unheated	IR- at 130 °C	IR- at 150 °C	Unheated	IR- at 130 °C	IR- at 150 °C
C18:2(6)	2.24 ^c^	3.89 ^b^	4.22 ^b^	5.16 ^a^	2.43 ^c^	2.38 ^c^	2.68 ^c^
C18:3	0.31 ^b^	0.32 ^b^	0.40 ^a^	0.42 ^a^	0.33 ^b^	0.34 ^b^	0.41 ^a^
C22:5(3)	0.12 ^ab^	0.09 ^b^	0.12 ^ab^	0.15 ^a^	0.12 ^ab^	0.11 ^ab^	0.12 ^ab^
Omega-3	0.43 ^a^	0.41 ^b^	0.52 ^a^	0.57 ^a^	0.45 ^b^	0.45 ^b^	0.52 ^a^
Omega-6	2.76 ^c^	4.32 ^b^	4.83 ^b^	5.82 ^a^	2.95 ^c^	2.81 ^c^	3.21 ^c^

Mean values followed by the same letter in the same row are not significantly different at *p* < 0.05. (Adapted with permission from Shariati-Ievari et al. [116], published by John Wiley and Sons, 2016).

**Table 6:** Performance of IR-treated lentil flour in processed meat products.

Meat Product and Lentil Inclusion Rate and IR Treatment Conditions	Control Binder	Product Performance of IR-Treated Lentil Flour in Comparison with Specific Control Binder				Reference
		Effect on Cooking Properties and Proximate Composition	Effect on Textural Properties	Effect on Oxidative Stability	Effect on Consumer Acceptability (Sensory Score of Cooked Products Compared with Control)	
Beef burger 6% and 12% lentil flour, tempering to 15% moisture, IR heating 130–135 °C	No binder	↓ cook loss ↓ diameter reduction ↓ thickness reduction ↑ moisture retention NSD fat retention NSD composition	↓ shear force ↓ hardness ↓ cohesiveness ↓ springiness	↓ TBARS at day 7 at 4 °C ↑ redness (a*) at day 7 at 4 °C	↑ juiciness ↑ tenderness ↓ cohesiveness NSD flavor acceptability NSD off-flavor ↑ overall acceptability	[16]
	6, 12% untreated lentil flour	NSD cooking yield NSD diameter reduction NSD thickness reduction NSD moisture retention NSD fat retention NSD composition	NSD shear force ↑ hardness NSD cohesiveness NSD springiness		NSD juiciness NSD tenderness NSD cohesiveness ↑ flavor acceptability ↓ off-flavor ↑ overall acceptability	
	6% toasted wheat crumb		NSD shear force NSD hardness NSD cohesiveness ↑ springiness		NSD juiciness NSD tenderness NSD cohesiveness ↑ flavor acceptability NSD off-flavor NSD overall acceptability	
	6% wheat flour		NSD shear force ↑ hardness NSD cohesiveness ↑ springiness		NSD juiciness ↓ tenderness NSD cohesiveness NSD flavor acceptability NSD off-flavor NSD overall acceptability	
Beef burger 6% lentil flour, tempering to 16 and 23% moisture, IR heating 115, 130, 150, 160 °C	No binder	NSD composition		↑ redness (a*) value on day 4 in retail display at 4 °C ↓ TBARS on day 4 in retail display at 4 °C ↓ TBARS at month 4 in freezer at −35 °C		[117]
	6% untreated lentil flour					
	6% toasted wheat crumb					
Beef burger 6% lentil flour, no tempering, natural moisture content, IR heating 130 and 150 °C	No binder	↓ cook loss ↓ drip loss ↓ shrinkage	↓ shear force			
	6% untreated lentil flour	NSD cook loss NSD drip loss NSD shrinkage NSD composition	NSD shear force		↑ appearance ↑ aroma ↑ flavor ↑ texture ↑ overall acceptability	[116]
	6% untreated chickpea flour					
	6% IR-treated chickpea flour				NSD appearance NSD aroma NSD flavor NSD texture NSD overall acceptability	
Beef burger 6% lentil flour, tempering 23% moisture, IR heating 150 °C	No binder	NSD cook loss NSD drip loss NSD shrinkage NSD composition		↑ redness (a*) on day 3 at 4 °C ↓ TBARS on day 3 stored at 4 °C ↑ redness (a*) at week 12 at −18 °C ↓ TBARS at week 12 at −18 °C		[18]
	6% untreated lentil flour					
	6% toasted wheat crumbs					
Chicken bologna 6% commercial IR heat-treated flour (NutraReady™, Saskatoon, SK, Canada)	No binder	NSD cook loss ↓ expressible ↓ purge loss	↑ shear stress NSD shear strain ↑ hardness ↓ cohesiveness ↓chewiness NSD springiness	↓ TBARS at week 8 at 4 °C	↓ color acceptability ↓ juiciness ↑ graininess NSD overall flavor NSD foreign flavor ↑aftertaste NSD overall acceptability	[81]
Chicken wiener sausage 4, 6, 8% Commercial IR heat-treated flour (NutraReady™, Saskatoon, SK, Canada)	No binder	NSD cook loss ↓ expressible moisture ↓ purge loss	NSD hardness ↓ cohesiveness NSD chewiness NSD springiness	NSD TBARS at week 6 at 4 °C	NSD external color acceptability NSD internal color acceptability NSD texture NSD juiciness NSD flavor NSD overall acceptability	[81]
	1.75% Isolated soy protein with corn starch 4%	NSD cook loss NSD expressible moisture NSD purge loss	NSD hardness NSD cohesiveness NSD chewiness NSD springiness	NSD TBARS at week 6 at 4 °C		
Pork bologna 6% commercial IR heat-treated flour (NutraReady™, Saskatoon, SK, Canada)	No binder	NSD cook loss ↓ expressible moisture NSD purge loss NSD thickness reduction NSD moisture retention NSD fat retention	↑ shear stress NSD shear strain NSD hardness NSD chewiness	NSD TBARS NSD carbonyl NSD sulfhydryl at week 12 at −18 °C	↓ external color acceptability ↓ internal color acceptability ↓ purge color acceptability	[118]

NSD: no significant difference, ↑: increased, ↓: decreased, TBARS: thiobarbituric acid reactive substances.

## Data Availability

No new data were created or analyzed in this study. Data sharing is not applicable to this article.
